# The cardio-oncologic burden of breast cancer: molecular mechanisms and importance of preclinical models

**DOI:** 10.1007/s00395-024-01090-w

**Published:** 2024-12-02

**Authors:** J. Brauer, M. Tumani, N. Frey, L. H. Lehmann

**Affiliations:** 1 Department of Cardiology, University Hospital Heidelberg, Im Neuenheimer Feld 410, 69120 Heidelberg, Germany; 2 German Center of Cardiovascular Research (DZHK), Partnersite Heidelberg, Mannheim, Germany; 3 German Cancer Research Center (DKFZ), Heidelberg, Germany

**Keywords:** Cardio-oncology, BRCA1, BRCA2, Breast cancer

## Abstract

Breast cancer, the most prevalent cancer affecting women worldwide, poses a significant cardio-oncological burden. Despite advancements in novel therapeutic strategies, anthracyclines, HER2 antagonists, and radiation remain the cornerstones of oncological treatment. However, each carries a risk of cardiotoxicity, though the molecular mechanisms underlying these adverse effects differ. Common mechanisms include DNA damage response, increased reactive oxygen species, and mitochondrial dysfunction, which are key areas of ongoing research for potential cardioprotective strategies. Since these mechanisms are also essential for effective tumor cytotoxicity, we explore tumor-specific effects, particularly in hereditary breast cancer linked to BRCA1 and BRCA2 mutations. These genetic variants impair DNA repair mechanisms, increase the risk of tumorigenesis and possibly for cardiotoxicity from treatments such as anthracyclines and HER2 antagonists. Novel therapies, including immune checkpoint inhibitors, are used in the clinic for triple-negative breast cancer and improve the oncological outcomes of breast cancer patients. This review discusses the molecular mechanisms underlying BRCA dysfunction and the associated pathological pathways. It gives an overview of preclinical models of breast cancer, such as genetically engineered mouse models, syngeneic murine models, humanized mouse models, and various in vitro and ex vivo systems and models to study cardiovascular side effects of breast cancer therapies. Understanding the underlying mechanism of cardiotoxicity and developing cardioprotective strategies in preclinical models are essential for improving treatment outcomes and reducing long-term cardiovascular risks in breast cancer patients.

## Introduction

Breast cancer is the most common cancer affecting women worldwide [79, 175]. Despite environmental risk factors, such as age, smoking, obesity, or increased exposure to estrogen, up to 10% of all mamma-carcinoma are considered to be caused by pathogenic variants in cancer predisposition genes [110, 113, 127, 134]. Pathogenic variants of BRCA1 and BRCA 2 are the most frequent causes for hereditary breast cancer [20]. Primary and secondary prevention strategies have been established to prevent and detect mamma-carcinoma in an early stage. Women with hereditary breast cancer are often young compared to the non-hereditary group [201]. Specifically young women (age 35–65) with breast cancer (e.g., BRCA-related) who receive potentially cardiotoxic chemotherapy face significant cardio-oncological risks [111, 161]. This increased risk has two major reasons: (1) The patients have a longer survival and reach more frequently a survival time where long-time cardiovascular complications occur, leading to a higher incidence of cardiovascular death after a survival of 10 years [174]. (2) The incidence for triple negative breast cancer (TNBC) is higher in this population and would need a more intensive chemotherapy [63]. Novel therapies, such as immune checkpoint inhibitors, are currently in clinical testing to treat patients with TNBC. However, the ‘classical’ chemotherapy, including anthracyclines and human epidermal growth factor receptor 2 antagonists (HER2) in case of HER2 positive expression, are still the pillars of breast cancer therapy with a risk for acute and long-term cardiovascular toxicity. The review discusses molecular consequences of the BRCA mutations and pathological pathways related to cardiotoxicity. It gives an overview of toxic therapies and discusses suitable preclinical models for cancer and cancer therapy.

## Molecular mechanism of breast cancer and resulting drug targets

BRCA1 and BRCA2 are crucial for the DNA damage repair [53, 129]. They play an important role in the repair of double-strand breaks through homologous recombination (HR), activation of the cell cycle checkpoint and apoptosis [151, 173, 178]. Dysfunctional forms of BRCA genes are less capable to protect the genome against environmental and endogenous toxicity and could finally lead to tumorigenesis [61, 69, 96]. In the cardiovascular system, early preclinical studies highlight the molecular importance of BRCA1 for survival of cardiomyocytes [166].

### DNA repair mechanisms

The homologous recombination is one of the two major mechanisms to repair double-strand breaks (DBS). The repair pathway is activated exclusively in the late S and G2 phase of the cell cycle and needs an intact homologue sister chromatin as a DNA template for the repair [32]. BRCA 1 is phosphorylated and activated by ataxia–telangiectasia mutated (ATM) which coordinates the cellular response to double strand breaks [198]. Afterwards, BRCA1 binds to DSB through the abraxas–RAP80 macro-complex, which induces ubiquitination of histones at DNA of the DSB [192]. By cooperating with specific proteins, such as Mre11, Rad50, and Nbs1 (MRN) BRCA 1 forms a complex with CtIP, to promote 5′-end resection in the early steps of the synthesis-dependent strand annealing pathway of HR [33]. Both, RAD51 and RAD50 are also used as a readout for cardiotoxicity of anthracyclines [169, 200], which is known to interfere with a sufficient DNA repair mechanism in cardiomyocytes. Moreover, dysfunctional BRCA complexes are discussed to prime for cardiotoxicity in patients treated with anthracyclines [82, 154] and are known to prime for a decline in cardiac function upon anthracycline therapy in preclinical models [166, 169]. BRCA1 interacts with multiple proteins, such as PALB2 and BRCA2 to recruit RAD51, an essential mediator of homologous recombination (HR) [151]. A dysfunctional BRCA1 or BRCA2 breaks apart the PALB2–BRCA2 complex, which leads to the abrogated HR repair process in case of a double-strand break. This ultimately increases then the risk for the development of mammary and endometrial tumors [94]. BRCA1 also interacts with BACH1 and is involved in many other DNA repair mechanisms. Mutations of BACH1 or BRCA1 affect the DNA repair process negatively and also result in a higher risk for breast cancer [138]. BRCA2 is primarily involved in binding to RAD51 [159]. BRCA2 also interacts with PALB2, linking BRCA1 to BRCA2 in a cell cycle-dependent manner. Mutations in either BRCA2 or PALB2 reduce the HR repair capacity, thereby increasing the risk of breast cancer [182] and potentially prime for cardiotoxic events [82]. These insufficient DNA repair mechanisms are known to be deleterious in anthracycline toxicity and could therefore be a target for cardioprotective strategies [207].

#### BRCA and the risk to develop atherosclerosis

BRCA mutations may also interfere with the pathophysiology of atherosclerosis. It has already been shown that BRCA1/BRCA2-deficient cells are more sensitive to oxidative stress [51]. Furthermore, Shing et al. demonstrated that BRCA1 overexpression strongly attenuated the production of reactive oxygen species, upregulates endothelial NO synthase, phosphorylates Akt, and promotes the expression of vascular endothelial growth factor and finally leads to significantly less aortic plaque lesions and reduced macrophage infiltration in ApoE -/- mice fed a western diet [168]. Correspondingly, BRCA1 expression was reduced in the plaque region of human atherosclerotic carotid artery samples compared with the adjacent plaque-free area[168].

The cardioprotective role has been further investigated in BRCA1/2-knockout mice. BRCA genes protect cardiomyocytes from DNA damage, apoptosis and finally heart dysfunction [166]. In regard to atherosclerosis, the loss of BRCA1 in cardiomyocytes was associated with a reduction in regulating cardiac energy supply, promoting endothelial cell apoptosis and endothelial dysfunction [170]. Mechanistically, the loss of BRCA1 results in impaired DNA double strand break repair and an activation of p53-proapoptotic signaling pathway [166].

Insulin resistance (IR) and atherosclerosis are strongly connected. IR plays a significant role in the development and progression of atherosclerosis. Both conditions are frequently seen in metabolic disorders. IR contributes to endothelial dysfunction by impairing insulin signaling in endothelial cells and reducing the NO availability [120]. Furthermore, IR is associated with chronic inflammatory condition, dyslipidemia and high levels of ROS [148]. These factors promote the development and progression of atherosclerotic plaques, increasing the risk of cardiovascular events [131].

Interestingly, BRCA1/2 mutation carriers often have very low or very high levels of insulin-like growth factor 1 (IGF-1) in the serum compared to non-carriers, which is associated with an increased risk for insulin resistance [52, 93, 136]. IR is an important predictor for the development of type-II diabetes mellitus (T2DM), which is part of the metabolic syndrome. In addition, a higher risk of developing diabetes after the diagnosis of breast cancer for BRCA1/2 mutation carriers has been described [19].

The role of BRCA1/2 and other regulators of DNA repair in the pathophysiology of cardiovascular diseases is complex and not yet fully understood, but the evidence suggests that the genes are important to maintain cardiac and vascular health by promoting sufficient DNA repair, regulation of oxidative stress, and inflammation control. Further research is needed to clarify the exact mechanisms and clinical implications. Even before a potentially cardiotoxic therapy, there is evidence that patients with a BRCA mutation have a higher cardiovascular risk compared to non-carriers.

### Clinical consequences

Pathogenic variants of BRCA1 and BRCA2 are responsible for approximately 25% of all hereditary breast cancer cases [113]. Women with a pathogenic variant of BRCA 1 have a lifetime risk of 70–80% for the development of a mamma-carcinoma [86]. In addition to that, their lifetime risk for other tumor entities like ovarian-carcinoma is also significantly increased. BRCA1 mutated breast cancers are negative for estrogen receptor (ER), progesterone receptor (PR), and human epidermal growth factor receptor 2 (HER2) and are classified as TNBC [128]. In general, TNBC have a poorer prognosis compared to other breast cancer subtypes with a median survival of less than 24 months [31]. The subtype has also a higher possibility of early recurrence, especially within the first 3–5 years after diagnosis because of being more aggressive and the trend to grow and spread faster [68]. Due to the lack of the receptors, the treatment options are limited, because the tumor does not respond to hormonal therapies or HER2-targeted therapies. Therefore, chemotherapy is the primary treatment modality, which can be effective but often has severe side effects. New therapies, including PARP inhibitors and immunotherapy, are being investigated and have shown promising effects in clinical trials [68].

The lifetime risk for breast cancer with a pathogenic variant of BRCA2 is about 45–65% [10]. In contrast to BRCA1 mutation carriers, BRCA2 mutated breast cancers have similar pathological features to sporadic breast cancer. There has been no correlation between BRCA2 and TNBC.

### Preclinical models for breast cancer research: an overview

Breast cancer research has greatly benefited from a variety of preclinical models, which are essential for understanding the described disease mechanisms, testing new therapies, and developing personalized treatment strategies. These models range from in vitro and ex vivo systems (listed in Table 1) to sophisticated in vivo models (listed in Table 2), each with its unique advantages and limitations.

**Table 1:** Preclinical in vitro and ex vivo models of breast cancer

Model	Description	Main advantages	Main disadvantages	References
Patient-derived tumor xenografts (PDXs)	Transplantation of tumor pieces from patients into immunodeficient mice (e.g., NOD–SCID, NSG); cellular and histopathological structures of the original tumors are preserved	More accurate predictions of therapeutic responses, preservation of tumor architecture and genetic characteristics	Requires highly immunodeficient mice	[205]
Tumor organoids	3D cultures of primary tumor cells	Preservation of the heterogeneous composition of tumors; useful for drug testing and personalized treatment strategies	May not fully replicate the complexity of the tumor environment	[205]
Extracellular matrix (ECM) models	2D cultures (e.g., MCF10A, MCF10DCIS.com) and 3D cultures (e.g., 3D Matrigel) with purified ECM components or decellularized tissue scaffolds	Natural environment for studying cell behavior and invasiveness, more realistic in vitro model of DCIS (ductal carcinoma in situ)	Does not fully represent the complexity of the in vivo tumor environment	[21]
Co-culture systems	Combination of epithelial cells with stromal fibroblasts or immune cells	Increased complexity of in vitro models, useful for studying tumor-stroma and immune cell interactions	Complex setup and maintenance	[21]
Ex vivo microfluidic systems	Murine and patient-derived organotypic tumor spheroids with autologous stromal/immune cells	Preserve tumor-infiltrating myeloid and lymphoid subpopulations, rapid treatment results	High technical complexity	[2]
Perfusion-based bioreactors	Preservation of viability and functional activity of breast cancer tissues through continuous media flow	Long-term survival and maintenance of tissue ecosystem, useful for evaluating targeted therapies	High technical complexity, potential ethical concerns	[2]

**Table 2:** Preclinical animal models of breast cancer

Model	Description	Model designations	Main advantages	Main disadvantages	References
Genetically Engineered Mouse Models (GEMMs)	Use of genetically modified mice to model various breast cancer types (e.g., luminal A, BRCA1-mutated TNBC, invasive lobular carcinomas)	MET1, MVT1, R3T	Produces orthotopic de novo breast cancer within a physiological tumor environment, reconstructs histopathological features	Expensive, time-consuming, low incidence of metastasis, low tumor mutation burden	[2] [202] [88]
Syngeneic Murine Models	Use of murine tumor cell lines transplanted into immunocompetent mice of the same strain	4T1, 66cl4, EMT6, E0771	Immunocompetent environment, valuable for immunotherapy studies	Low immunogenicity, may not fully mimic the human disease	[2, 132, 202]
Humanized Mouse Models	Reconstitution of immunodeficient mice with human cells to create a partially functional human immune system	BR1126, MDA-MB-231, MC1, BCM-4913/4664/5471	Study of human cancer cells in a relevant immunological context	Develops xenograft-versus-host disease, lack of essential cytokines for myeloid lineage differentiation	[2, 150, 194]
Metastasis Models	Use of human tumor cell lines and selection processes to study metastatic processes	MDA-MB-231, LM2.4	Study of metastatic processes and therapeutic interventions	May not fully capture the complexity of human metastatic disease	[92]
Ehrlich Tumor Model	Derived from a spontaneous murine mammary carcinoma, exists in solid tumor and ascites form	n/a	Evaluation of chemotherapeutic efficacy, study of tumor growth mechanisms, immunotherapy research	Species differences, tumor heterogeneity	[146]

#### Patient-derived tumor xenografts (PDXs) and tumor organoids

Patient-derived tumor xenografts (PDXs) and tumor organoids are essential in preclinical models for personalized medicine of breast cancer [205]. PDXs involve transplanting small pieces of tumors from cancer patients into highly immunodeficient mice, such as NOD–SCID and NSG mice, which preserve the cellular and histopathological structures of the original tumors. Cytogenetic analyses have shown that PDXs maintain the genomic and gene expression profiles of the patient tumors, providing predictions of possible therapeutic responses.

Tumor organoids, on the other hand, are three-dimensional cultures of primary cancer cells that closely mimic the morphological, genetic, and epigenetic features of the parent tumors [209]. These organoids preserve the heterogeneous composition of the original tumor and offer promising opportunities for drug screening and personalized treatment strategies. They enable the creation of large biobanks with clinically relevant materials, facilitating chemical discovery and the identification of optimal treatment strategies for individual patients.

##### Extracellular matrix (ECM) models and co-culture systems

Brock et al. discussed various in vitro models for studying the transition of ductal carcinoma in situ (DCIS) to invasive ductal carcinoma (IDC) [21]. Two-dimensional (2D) cell culture models, such as MCF10A cells and MCF10DCIS.com cells, serve as baselines for comparison with transformed and invasive cell lines. These models are cost-effective and easy to use but lack the complexity of the tumor environment. Three-dimensional (3D) cell culture models, including 3D Matrigel cultures and organotypic cultures, aim to replicate the tumor microenvironment more accurately by supporting cell–cell and cell–matrix interactions. These models provide a more realistic in vitro model of DCIS by co-culturing tumor cells with stromal cells, better mimicking DCIS architecture.

Co-culture systems, such as epithelial–stromal co-cultures and immune cell co-cultures, further enhance the complexity of in vitro models by combining epithelial cells with stromal fibroblasts or immune cells. These systems are used to study the influence of stromal components and immune cells on DCIS behavior and invasiveness. Cancer-cardiomyocyte co-cultures were used to investigate intercellular communication [157] to study the clinical phenomenon of cardiac wasting [9] but not to assess toxic potential of drugs in parallel to cytotoxic effects on tumor cells.

##### Genetically engineered mouse models (GEMMs)

Genetically engineered mouse models (GEMMs) have become pivotal in breast cancer research, offering advanced tools to replicate various aspects of human breast cancer with greater accuracy. These models have largely replaced syngeneic models, particularly with the advent of human-directed targeted therapies [156]. GEMMs leverage genetic engineering to allow direct gene editing in germline and/or somatic cells, with somatic cell-derived GEMMs potentially providing a more precise representation of human tumors [2].

Notable GEMMs include those for the luminal A subtype, BRCA1-mutated TNBC, and invasive lobular breast cancer [2]. For instance, the BRCA1-mutated TNBC model represents TNBC with BRCA1 mutations, characterized by high genomic instability but taking considerable time to develop [41, 162].

GEMMs offer significant advantages, including the ability to form orthotopic de novo breast cancer within a physiological tumor microenvironment and to recapitulate the histopathological features of human breast cancer. However, they also face considerable limitations: they are costly, time-consuming, have relatively low metastatic incidence, low tumor mutational burden, and often exhibit supraphysiological levels of modified transgenes, raising questions about their clinical relevance.

Furthermore, GEMMs display diverse proliferative, angiogenic, and immunogenic profiles, which impact their utility in studying immune responses and the efficacy of ICIs [139]. Despite their potential, GEMMs are less exploited in ICI testing compared to syngeneic models, primarily due to these limitations and the need for extensive expertise and infrastructure [2].

##### Syngeneic murine models

Syngeneic murine models of breast cancer involve grafting murine tumor cell lines into immunocompetent mice of the same strain. These models provide valuable insights into breast cancer biology, metastasis, and responses to immunotherapy [2].

The 4T1-model for instance originates from the mammary fat pad of BALB/c mice and mimics aggressive human breast cancer metastasis by spreading spontaneously to various organs [11]. It is characterized by low immunogenicity, posing challenges for the efficacy of immune checkpoint inhibitors (ICIs) when used alone [37, 89, 144].

66cl4, 168FARN, 67NR, and 4TO7 models vary in their metastatic potential [132, 202]. These models provide a range of options for studying different aspects of metastasis in breast cancer. EMT6 is partially responsive to anti-CTLA-4 and anti-PD-L1 therapies, making it suitable for investigating immune checkpoint inhibitor responsiveness and interactions with the tumor microenvironment E0771, derived from C57BL/6 mouse mammary adenocarcinoma, exhibits moderate-to-high metastatic potential, primarily targeting the lungs [144, 152]. It is commonly used in studies focusing on immunotherapy, providing insights into treatment efficacy in preclinical settings.

These syngeneic models offer crucial advantages like immunocompetent environments for studying tumor–immune interactions and evaluating novel therapies, particularly in the context of immunotherapy.

##### Humanized murine models

Humanized mouse models have emerged as a critical tool in cancer research to address the limitations of syngeneic and genetically engineered mouse models (GEMMs), particularly due to the lack of human targets and inherent interspecies immunological differences [2]. Significant efforts have been made to humanize the murine immune system, enabling the study of human cancer cells in a more relevant immunologic context.

One such model is the immunoavatar model, where immunodeficient mice are reconstituted with human peripheral blood mononuclear cells (PBMCs). This allows the study of human cancer cell lines or primary patient-derived tumors. However, this model often develops human xenograft versus host disease a few weeks post-engraftment due to MHC mismatch between mouse and human T lymphocytes [156].

Alternatively, fully immunocompromised mice, such as NOD scid gamma (NSG) mice, are engrafted with human hematopoietic stem and progenitor cells (HPSCs), giving rise to a partially functional human immune system [194]. Human cord blood cells are commonly used for this purpose due to their accessibility and superior engraftment rates compared to adult bone marrow cells [25]. However, these models are limited by the lack of essential cytokines needed for the maturation and differentiation of myeloid lineages.

Despite these advancements, humanized models still face challenges, including the complexity of sequential human immune/tumor cell engraftment. However, ongoing efforts to optimize these models are expected to lead to a new generation of more humanized models, which could revolutionize cancer and cardio-oncological research (e.g., cardiotoxicity of immune therapies) [2].

##### Preclinical models for metastasis

Kerbel et al. focused on preclinical models for metastasis, emphasizing human tumor cell lines and selection processes [92]. The primary cell line used is MDA-MB-231, derived from a TNBC patient [121]. The selection process involves orthotopic transplantation of MDA-MB-231 cells into the mammary fat pad of mice to establish primary tumors, followed by surgical resection to allow microscopic metastases time to develop. Metastases are isolated and cultured to establish new cell lines, and serial selection is performed to select variants with enhanced metastatic potential, resulting in more aggressive metastatic variants.

##### Ehrlich tumor model

The Ehrlich tumor model, derived from a spontaneous murine mammary adenocarcinoma, which exists in two forms: solid tumor and ascitic form [49, 146]. This model exhibits characteristics similar to human breast cancer, such as high mitotic activity, pleomorphism, and invasiveness. It is frequently used to evaluate the efficacy of chemotherapeutic agents, study the molecular and cellular mechanisms of tumor growth and metastasis, and investigate the effects of immune modulators and vaccine strategies due to its immunogenic properties. Despite limitations like species differences and tumor heterogeneity, the Ehrlich tumor model remains a valuable tool in breast cancer research for drug testing, mechanism studies, immunotherapy research, radiotherapy, and anti-angiogenesis studies.

### Conclusion

The diversity of preclinical models in breast cancer research allows for comprehensive studies of tumor biology, metastasis, and therapeutic responses. In vitro and ex vivo models, such as PDXs, tumor organoids, ECM models, and co-culture systems, offer detailed insights into cellular interactions and personalized treatment strategies. Animal models, including GEMMs, syngeneic murine models, and metastasis models, provide complex in vivo environments to study disease progression and therapeutic interventions. Many of these models would be valuable tools for cardio-oncology.

## Treatment of breast cancer

The molecular changes underlying cancer therapy-associated cardiovascular toxicity involve numerous pathways, which are reviewed extensively elsewhere [155]. This section is only able to cover a number of prominent pathways that are currently discussed as potential therapeutic targets and focuses on the preclinical mouse models that are currently in use to investigate therapy-related cardiotoxicity. While there is literature on rat- and large animal models [56, 141], we focus mainly on mouse models due to their applicability in transgenic and knock-in/-out studies.

### Anthracyclines

Anthracyclines are one of the most effective agents for both early and advanced breast cancer. However, the potential benefit is limited to the cardiotoxicity which involves a large number of pathological molecular events, and which is related to the cumulative dose [77, 155]. To minimize cardiotoxicity while maintaining the same antitumor effect liposomal doxorubicin was developed. Some trials have already proved the antitumor efficacy and reduced cardiotoxicity of liposomal doxorubicin [199].

The effects of anthracyclines include DNA intercalation, inhibition of topoisomerase II and the formation of reactive oxygen species, which leads to DNA damage and apoptosis. Topoisomerase II is crucial for DNA replication and transcription. The inhibition prevents the re-ligation of DNA strands. Doxorubicin (DOX) also alters the Bcl-2/Bax ratio, leading to the activation of downstream caspases and ultimately resulting in apoptosis [7].

The toxic effect of anthracyclines unfolds in mitochondria, in the cytosol and in the cell nucleus [102]. Doxorubicin leads to changes in the expression of mitochondrial genes and to the generation of intramitochondrial superoxides, which ultimately cause loss of the organelle through increased mitophagy. The mitochondrial redox reaction is directly disturbed, which is accompanied by an increase of reactive oxygen species (ROS). In vitro studies reveal that the direct supplementation of nicotinamide adenine dinucleotide (NAD +) attenuates the toxic effect [177]. However, antioxidants in animal models have not yet shown strong cardioprotective effects. Interestingly, various studies highlight both the damaging and beneficial roles of ROS in heart disease, while also exploring potential therapeutic strategies that target ROS for cardioprotection. This inherent contradiction likely contributes to the failure of clinical trials and hinders the development of new cardioprotective strategies that rely only on eliminating ROS [57, 75, 76]

Cytosolic mechanisms, e.g., an altered Ca2 + , balance through regulation of the expression and activity of the sarcoplasmic/endoplasmic reticulum Ca2 + ATPase (SERCA2) and dysregulation of mitochondria, autophagy and dysregulation of kinases/phosphatases (e.g. the Ca2 + /calmodulin-dependent proteinkinase II, CaMK2 and Phosphoinositide 3-Kinase Gamma, PI3Kγ) are also involved [73, 105, 184].

Studies have also shown an activation of the proapoptotic regulator p53 has damaging effects of anthracycline treatment [206]. Interestingly, low-dose DOX treatment leads to an activation of p53 as part of the endogenous repair mechanism and protects the heart [103].

Inhibition of ROS generation, proapoptotic proteins or genetic deletion of the topoisomerase IIb or PI3Kγ reduces the cardiotoxic effect in preclinical models. Unfortunately, a clinical translation of these findings has not yet been achieved. However, a new anti-apoptotic substance showed promising protection in preclinical settings with the clear goal of a timely clinical translation [7].

#### Anthracycline-induced cardiotoxicity

The incidence of anthracycline-induced cardiotoxicity varies widely, with heart failure rates reported between 3 and 26%, depending on cumulative dose and individual patient risk factors [38]. Higher cumulative doses, particularly above 400 mg/m^2^, significantly increase the risk [176]. Other risk factors include age, pre-existing cardiovascular conditions, and the concurrent use of other cardiotoxic agents [74, 181]. Regular cardiac monitoring using echocardiography or MRI, along with biomarkers such as troponins and natriuretic peptides, are essential for early detection in patients at risk [108, 183, 203]. Dexrazoxane has been identified as a cardioprotective agent to mitigate these effects [29].

##### Preclinical models

Preclinical models have been pivotal in understanding the mechanisms of anthracycline-induced cardiotoxicity. Both large and small laboratory animals were used to analyze the anthracycline-induced cardiomyopathy further.

The cardiovascular system and the anatomical structures of large animals such as pigs and monkeys are similar to that of humans. Similar changes in the myocardium are observed during and after the administration of anthracyclines [47]. However, housing and maintenance of large animals are expensive and the techniques such as genetic manipulations, blood sampling and intravenous administration are more complex than in small animals. In addition, the induction of a cardiac phenotype takes more time in large animals [149].

The most commonly models used nowadays are rats and mice. In general, the experiment can be divided into short- and long-term experiments. In the short-term experiments animals receive one or up to six injections within 1 week with high doses to analyze the acute cardiotoxicity, while doses of 1–5 mg every week for 2–12 weeks are used for the long-term trials [26, 124]. Low-dose long-term experiments are suitable for analyzing the chronic cardiotoxicity effect of anthracyclines [27, 165]. Earlier, intravenous(IV) administration through the tail vein was used for establishing a DOX cardiomyopathy model. Nowadays, intraperitoneal (IP) method of DOX administration is mainly used due to the easy handling of large numbers of animals. Doxorubicin significantly reduces body, heart and left ventricular weight 1 week after stopping DOX [83]. Interstitial fibrosis of the left ventricle progressively increases after the end of DOX administration. Details about the most commonly used mouse models are summarized in Table 3 and current large animal models in Table 4.

**Table 3:** Preclinical mice models for anthracycline-induced cardiomyopathy

Authors	Dose	Cumulative dose	Route of administration	Monitoring cardiotoxicity	References
Preclinical models for chronic cardiotoxicity					
Forssen and Tokes (1981), Kanter et al. (1985), van Acker et al. (1996)	4–5 mg/kg	20–40 mg/kg	IV	Histology	[49, 92, 187]
Vandenwijngaert et al. (2017)	2 mg/kg	20 mg/kg–24 mg/kg	IP	Echo + histology	[189]
Sahu et al. (2016), Pillai et al. (2017), Díaz-Guerra et al. (2024)	5 mg/kg	15 mg/kg–25 mg/kg	IP	Echo, CK-MB, LDH, ALS, AST, histology	[40, 141, 154]
Hullin et al. (2018), Gioffré et al. (2019)	4 mg/kg	20 mg/kg–24 mg/kg	IP	Echo, troponin	[60, 82]
Preclinical models for acute cardiotoxicity					
Mostafa et al. (2000), Abd-Allah et al. (2002), Hullin et al. (2018), Pei et al. (2016)	15 mg/kg	Once	IP	Echo, histology	[1, 82, 120, 138]
Li et al. (2006), Neilan et al. (2006), Vandenwijngaert et al. (2017)	20 mg/kg	Once	IP	Echo, histology, troponin	[104, 126, 189]
Preclinical models for genetic modified mice for breast cancer and cardiotoxicity					
Singh et al. (2012)	10–20 mg/kg	Once	IP	Cardiomyocyte-specific BRCA2 knock-out mice	[169]

Modified from Podyacheva et al. [141] *IV* intravenous, *IP* intraperitoneal

**Table 4:** Preclinical large animal models for anthracycline-induced cardiomyopathy in large animals

Authors	Animal	Dose	Route of administration	Monitoring cardiotoxicity	References
Galán-Arriola et al. (2022)	Pig	0.45 mg/kg	IC	CMR, histology, immunostaining, protein expression, RNA, capillary density	[56]
Galán-Arriola et al. (2019)	Pig	0.45 mg/kg	IC	CMR, histology	[55]
Galán-Arriola et al. (2021)	Pig	0.45 mg/kg	IC	CMR, TEM, histology, protein expression, mtDNA	[57]
Mester-Tonczar et al. (2023)	Pig	60 mg/m2 body surface are	IV	RNA Sequencing	[114]
Nakata et al. (2024)	Female Yucatan miniature swine	2 mg/kg	IV	CMR, histology, cardiac biomarkers in serum	[123]
Nakamori et al. (2019)	Swine	1.6–2.4 mg/kg	IV	CMR, histology,	[122]

*CMR* cardiac magnetic resonance, *IC* intracoronary, *IV* intravenous, *IP* intraperitoneal, *mt* mitochondrial, *TEM* transmission electron microscopy

### HER2-targeted therapies

HER2-targeted therapies significantly improve outcomes in HER2-positive cancers but are associated with varying degrees of cardiotoxicity [87]. The primary manifestation of cardiotoxicity is a decline in LVEF and heart failure.

#### HER2-induced cardiotoxicity

Trastuzumab-induced cardiotoxicity varies widely in clinical trials, ranging from 2 to 28%. Pertuzumab, when used in combination with trastuzumab, does not significantly increase cardiotoxicity [163, 179]. Lapatinib generally has a lower incidence of cardiotoxicity, while trastuzumab emtansine (T-DM1) has a relatively favorable cardiotoxicity profile with lower rates of cardiac dysfunction compared to trastuzumab [28, 143]. Identified risk factors for cardiotoxicity include pre-existing cardiovascular disease, age, hypertension, diabetes, and previous anthracycline therapy [6, 84]. Routine cardiac monitoring and the use of cardioprotective medications are recommended upon detection of cardiotoxicity.

##### Preclinical models

Preclinical models, including in vitro studies and animal models, have provided significant insights into the mechanisms of HER2 inhibitor-induced cardiotoxicity. Rodent models, such as those with cardiomyocyte-specific HER2 deletion, exhibit dilated cardiomyopathy, underscoring HER2's importance in cardiac health [133]. These models demonstrate that HER2 inhibition leads to oxidative stress, mitochondrial dysfunction, and impaired cardiomyocyte survival [44]. Inflammation and fibrosis are also implicated in the progression of cardiotoxicity [60]. Preclinical studies with beta-blockers and ACE inhibitors have shown the importance of cardioprotective agents to reduce cardiac damage [130]. The common cumulative dose of trastuzumab in preclinical models ranges between 10 mg/kg and 60 mg/kg and is administered by intraperitoneal injection. Currently used models are listed in Table 5**.**

**Table 5:** Preclinical mice models for HER2 + -induced cardiomyopathy

Authors	Dose	Frequence	Cumulative dose	Route of administration	Monitoring cardiotoxicity	References
Trastuzumab						
Ye at. (2023)	2,5 mg/kg	Twice a week for 4 weeks	10 mg/kg	IP	Echo, troponin, histology	[204]
Min et. al (2023), Mohan et al. (2016), El Zarrad et. al (2013)	10 mg/kg	Once per week for 6 weeks/every day for 6 days	60 mg/kg	IP	Echo, histology, intracellular calcium measurement	[46, 115, 117]
Wei et al. (2023), Quagliariello et al. (2019)	10 mg/kg	Once per day	40 mg/kg	IP	Echo, histology, myocardial enzymes levels	[145, 195]
Andreata et al. (2023)	5 mg/kg	Twice per week	25 mg/kg	IP	Histology, apoptotic assays	[8]
Pertuzumab						
Mohan et al. (2016)	10 mg/kg	every day for 6 days	60 mg/kg	IP	Echo, Histology	[117]

*IP* intraperitoneal

### Combined cardiotoxicity of anthracyclines and HER2-targeted therapies

#### Clinical data

Combining anthracyclines with HER2-targeted therapies, particularly trastuzumab, exacerbates cardiotoxic effects [106]. The risk of heart failure and LVEF decline is significantly higher when these agents are used together. Strategies to mitigate these risks include dose adjustments, treatment interruptions, and the use of cardioprotective agents such as beta-blockers and ACE inhibitors [172].

#### Preclinical models

Preclinical studies have shown that combined treatment with anthracyclines and HER2 inhibitors results in synergistic cardiotoxic effects. For instance, rodent models treated with both trastuzumab, and anthracyclines show exacerbated cardiac damage, reflecting clinical observations [195]. These models are crucial for developing predictive models and identifying biomarkers for early detection of cardiotoxicity. Information about most recent features of mouse models is summarized in Table 6.

**Table 6:** Preclinical mice models for HER2 + and anthracycline-induced cardiomyopathy

Authors	Dose	Frequence	Cumulative dose	Route of administration	Monitoring cardiotoxicity	References
Wei et al. (2023)	Doxorubicin 15 mg/kg Trastuzumab 15.75 mg/kg	Once	15 mg/kg and 15.75mg/kg	IP	Echo, histology, CK, CK-MB, troponin	[195]
Hoffmann et al. (2021)	Doxorubicin 5 mg/kg Traszuzumab 4 mg/kg	Every week for 4 months	90 mg/kg and 64 mg/kg	IP	Echo, histology	[78]
Nicol et al. (2021)	Doxorubicin 4 mg/kg Trastuzumab	Each 6 injections within 2 weeks	24 mg/kg and 10 mg/kg	IP	Echo, troponin	[126]
Guenancia et al. (2016)	Doxorubicin 6 mg/kg Trastuzumab 10 mg/kg	Once	6 mg/kg and 10 mg/kg	IP	Echo, histology, troponin	[65]
Telles-Langdon et al. (2024), Asselin et al. (2020)	Doxorubicin 8 mg/kg Trastuzumab 3 mg/kg	Weekly for 3 weeks	24 mg/kg and 12 mg/kg	IP	Echo, histology	[12, 180]

*IP* intraperitoneal

### Radiation toxicity

**Fig. 1:**
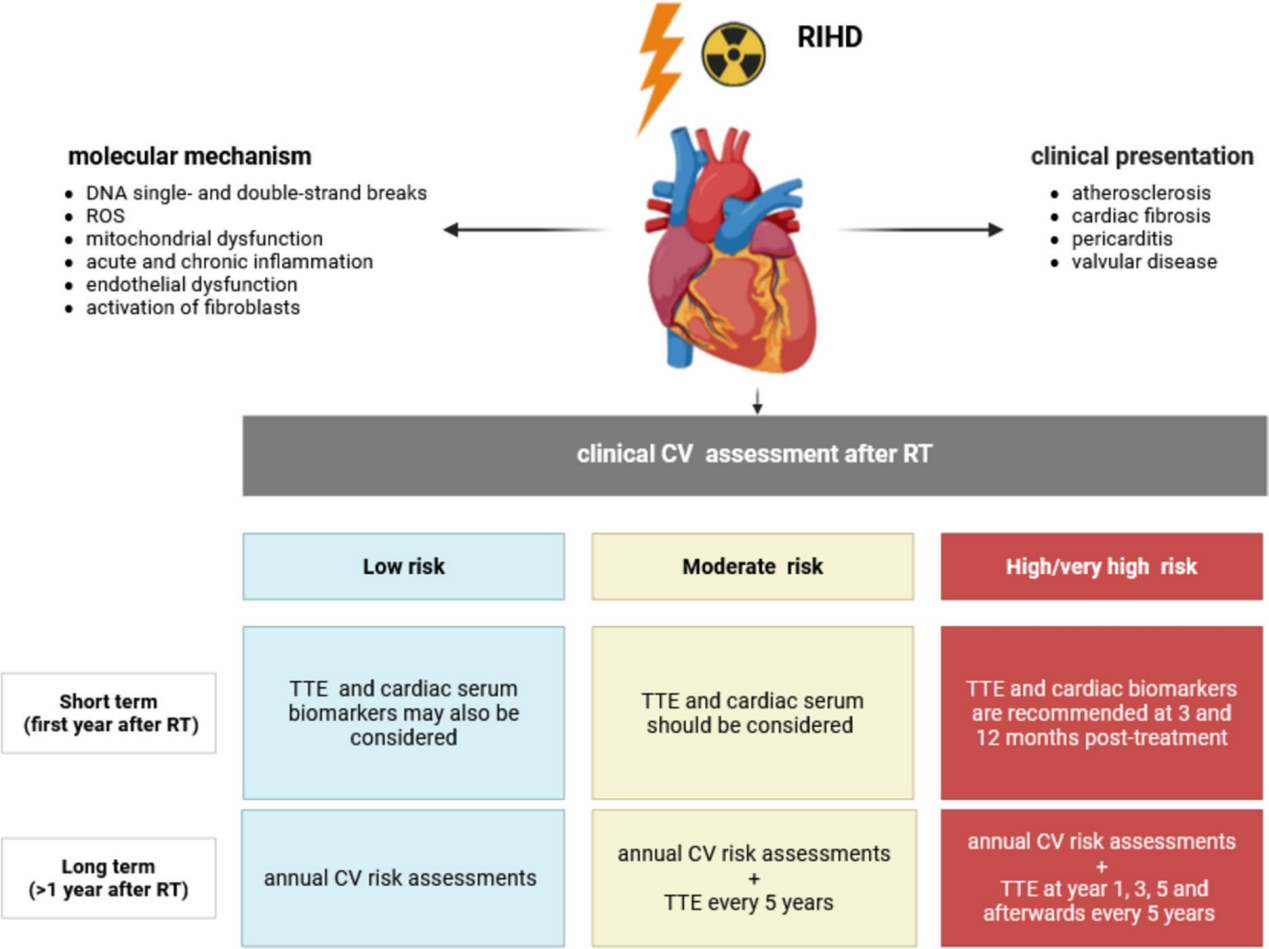
Schematic overview of RIHD (radiation-induced heart disease) and clinical CV (cardiovascular) assessment after RT (radiotherapy). ROS: reactive oxygen species; TTE: transthoracic echocardiography

Radiotherapy (RT) is a common and effective treatment modality in breast cancer, recommended in various clinical scenarios to improve local control of the disease and overall survival. However, radiotoxicity in the context of mamma-carcinoma represents a significant concern due to the adverse effects of radiotherapy on healthy tissues like the heart. Radiation-induced heart disease (RIHD) refers to a spectrum of cardiovascular complications like atherosclerosis, myocardial fibrosis, pericarditis and valvular disease after radiation (Fig. 1).

#### Pathophysiology or radiation-induced heart disease

The pathophysiology of RIHD is commonly associated with endothelial damage, edema, pericardial disease, and perfusion deficits [193]. The molecular mechanisms underlying RIHD are complex, involving multiple cellular and molecular pathways.

Ionizing radiation directly damages cellular DNA, resulting in single and double-strand breaks, which may ultimately trigger apoptosis of cardiomyocytes and endothelial cells [4]. In addition, radiation stimulates the overproduction of ROS, leading to oxidative stress and further damage, particularly to endothelial cells [15]. Endothelial injury is considered the primary factor in myocardial damage post-radiation, as these cells are highly sensitive to ionizing radiation [18, 101]. Although endothelial cells can regenerate, capillary network damage appears to be irreversible, which may compromise the myocardial blood supply [62].

Endothelial damage also activates inflammatory pathways, leading to immune cell infiltration and the release of pro-inflammatory cytokines. Changes in blood coagulation and platelet function are also observed, notably an increase in von Willebrand factor (vWF) production by endothelial cells [16, 85]. Elevated vWF levels enhance platelet adhesion, promoting thrombosis in capillaries [50]. This combination of thrombosis and reduced myocardial perfusion can lead to subtle myocardial ischemia.

ROS additionally impairs mitochondrial function and triggers acute inflammatory responses through damaged cells releasing pro-inflammatory signals attracting immune cells [171]. A persistent inflammatory response may ensue long-term, contributing to chronic endothelial dysfunction. This inflammation, along with endothelial damage, promotes the formation of atherosclerotic plaques and increases the risk of plaque rupture [80].

Radiation impacts not only large coronary vessels but also damages the smaller microvascular system within the myocardium, leading to microvascular disease [191]. Moreover, radiation stimulates fibroblasts and myofibroblasts to produce extracellular matrix (ECM) components, leading to fibrosis. This fibrosis stiffens both the myocardium and the pericardium, further impairing cardiac function. Fibrosis in radiation-induced heart disease (RIHD) not only affects the myocardium but can also extend to the vasculature, leading to significant perfusion deficits. This process impairs coronary blood flow by stiffening the walls of both large coronary arteries and smaller microvascular networks [90].

Radiation-induced endothelial damage increases capillary permeability, allowing fluid to leak into surrounding tissues. This fluid accumulation in the interstitial spaces can manifest as myocardial or pericardial edema, which impairs heart function and reduces cardiac output, potentially progressing to heart failure.

The pericardium is particularly vulnerable to inflammation and fibrosis following radiation exposure. Clinically, radiation-induced pericarditis may present in four stages: acute pericarditis, chronic pericarditis, fibrinous pericarditis, and the final stage of constrictive pericarditis [190]. The development of pericarditis is largely driven by DNA damage and oxidative stress in pericardial cells, leading to acute inflammatory responses and tissue necrosis within the pericardial layers. In addition, RT-induced endothelial damage within the pericardial vasculature increases permeability, further promoting fluid accumulation. The incidence of morbidity is strongly linked to the radiation dose absorbed by the heart [160]. The interplay between endothelial damage, inflammation, and fibrosis makes RIHD a highly complex condition, affecting multiple cell types within the heart.

Studies have revealed that RIHD can occur even after lower radiation doses (mean cardiac doses of 3–17 Gy) in breast cancer patients [34]. After high doses of radiation > 30 Gy RIHD can occur within the first 2 years after exposure. Patients with cardiovascular comorbidities, pre-existing cardiovascular disease and younger age have a higher risk to develop a RIHD [66, 147]. Moreover, the incidence of RIHD is increased in patients receiving RT of a high dose combined with anthracycline‑based chemotherapy [98].

#### Risk stratification and surveillance after radiotherapy

The heart is recognized as a radiosensitive "organ at risk" during radiotherapy (RT), and radiation exposure to the heart should be minimized as much as reasonably possible, as no dose can be considered completely “safe” (Table 7).

**Table 7:** General recommendations for cardiovascular (CV) surveillance of radiotherapy-treated (RT) patients based on the current guidelines of the ESC [108]

Cardiovascular toxicity risk	Dose and additional treatment	CV assessment after RT
Low risk	< 5 Gy MHD	May be considered
Moderate risk	5–15 Gy MHD < 5 Gy MHD + cumulative doxorubicin ≥ 100 mg/m2	Should be considered
High risk	> 15–25 Gy MHD 5–15 Gy MHD + cumulative doxorubicin ≥ 100 mg/m2	Recommended
Very high risk	> 25 Gy MHD > 15 Gy MHD + cumulative doxorubicin ≥ 100 mg/m2	Recommended

*Gy* grey, *MHD* mean heart dose

It is recommended to categorize the risk of RT-induced cardiovascular toxicity based on mean heart dose (MHD) rather than the prescribed dose, as the prescribed dose may not accurately represent the actual radiation exposure to the heart [109].

The timing of the first cardiovascular (CV) assessment after RT depends on the cardiovascular toxicity risk and whether cardiovascular toxicity (CTR–CVT) was diagnosed during treatment (Table 6). For patients receiving CV therapies (e.g., ACE inhibitors, beta-blockers, statins) for any CTR–CVT, especially cardiac dysfunction, clinical assessments, including ECG, echocardiography, and biomarkers, are advised at 3-, 6-, and 12-month post-treatment.

For the long-term surveillance after RT, the ESC recommends annual CV risk assessments and a cardiology referral if new CV symptoms develop independent of cardiovascular toxicity risk. In asymptomatic high and very high-risk patients a transthoracic echocardiography at years 1, 3 and 5 is recommended and afterwards every 5 years (Fig. 1).

#### Preclinical models

Preclinical models of radiation-induced heart disease (RIHD) are essential for understanding the mechanisms and developing treatments for this condition. Rodents, especially mice and rats, are commonly used in RIHD research [17, 71, 97]. Different genetically modified rodent models were developed to analyze different pathologies, such as atherosclerosis after radiation [54, 80, 158].

Howing et al. demonstrated that inflammatory and thrombotic pathways are upregulated after irradiation in ApoE −/− mice and underline the inflammatory response of atherosclerosis followed after irradiation [80]. Moreover, other mouse models describe the endothelial function in cardiac cell damage using p53 and p21 knock-out mice [100]. The author hypothesizes that the p53–p21 axis is able to prevent radiation-induced myocardial injury in mice. Partial heart irradiation has been used in different rodent models to identify potential regulatory pathways of the RIHD [42, 99, 167]. However, there are limitations in translating the results from rodent models to humans, due to the phylogenetic distance from humans and differences in the physiological responses to treatment regimens. Therefore, many studies have used rabbits to investigate the physiological response of the heart after irradiation. In general, rabbits serve as a good model to study RIHD except some limitations due to different heart rate, heart size and bodyweight in comparison to human. Limited numbers of studies [58]exist using canine models for heart radiation, despite the fact that canine coronary circulation have some similarities to ischemic hearts of human beings [24, 72]. The reason is most likely the lack of acceptance among the population for canine models, as well as stricter regulations and expensive housing and maintenance. Pigs and non-human primates present many similarities to RIHD of human beings, but only few studies exist due to the significantly higher costs compared to other animal models.

The combination of chemotherapy and radiation is a promising therapy strategy in breast cancer, which has been translated into an increase of survival rates [112]. Despite the promising efficacy of the combination of chemotherapeutic agents with radiotherapy, preclinical studies have shown an increase for adverse cardiac events like RIHD. The cardiac side effects often correlate with the cumulative dose [70]. Several retrospective studies have shown that combination therapy is associated with an increased risk of RIHD in NSCLC patients compared to those who only received radiation [13, 67]. Similar findings that chemoradiotherapy increases the risk of RIHD have been demonstrated in lymphoma [5, 187] and esophageal cancer [23, 197]. It has also been reported that radiation to the left chest has been linked to higher incidence of RIHD than radiation to the right chest [36].

A retrospective study of 5,260 breast cancer patients found that cardiovascular disease incidence was higher in those who received a radiation boost compared to those who did not [95]. Especially younger patients (< 50 years) showed a significantly higher incidence of ischemic heart disease (IHD) when they received a radiation boost. Derby et al. showed in a retrospective study of over 2000 women with breast cancer that the increase in the rate of ischemic heart disease is proportional to the mean dose to the heart and begins within a few years after exposure and continues for at least 20 years. The relative risk of coronary artery disease increased by approximately 7.4% per Gy of radiation to the heart [35]. In general, breast cancer patients are facing greater risk for RIHD because of their better life expectancy and survival rates compared to other cancer patients. Global longitudinal strain analysis could be useful to detect early subclinical cardiac dysfunction in breast cancer patients who underwent radiotherapy [64]. In summary, RIHD is a significant risk for breast cancer patients undergoing radiotherapy, particularly for those with left-sided breast cancer and those receiving higher radiation doses. A clinical follow-up and early detection through advanced imaging techniques are essential for breast cancer patients to initiate cardiovascular treatment and reduce long-term side effects.

### Immune checkpoint-inhibitors

Immunotherapy has revolutionized the treatment of multiple solid tumors in the last decade. In general, breast cancer is characterized by a low tumor mutation burden compared to other tumors and is therefore not considered as a highly immunogenic tumor. Nevertheless, different subtypes of breast cancer exist with different sensitivities to treatments. TNBC is considered the most immunogenic subtype of breast cancer with higher rates of PD-L1 expression on both tumor and immune cells [116]. Therefore, studies mainly focus on TNBC. The use of anti-PD1 and anti-PD-L1 as a single agent has shown only limited response rates [3, 40].

**Fig. 2:**
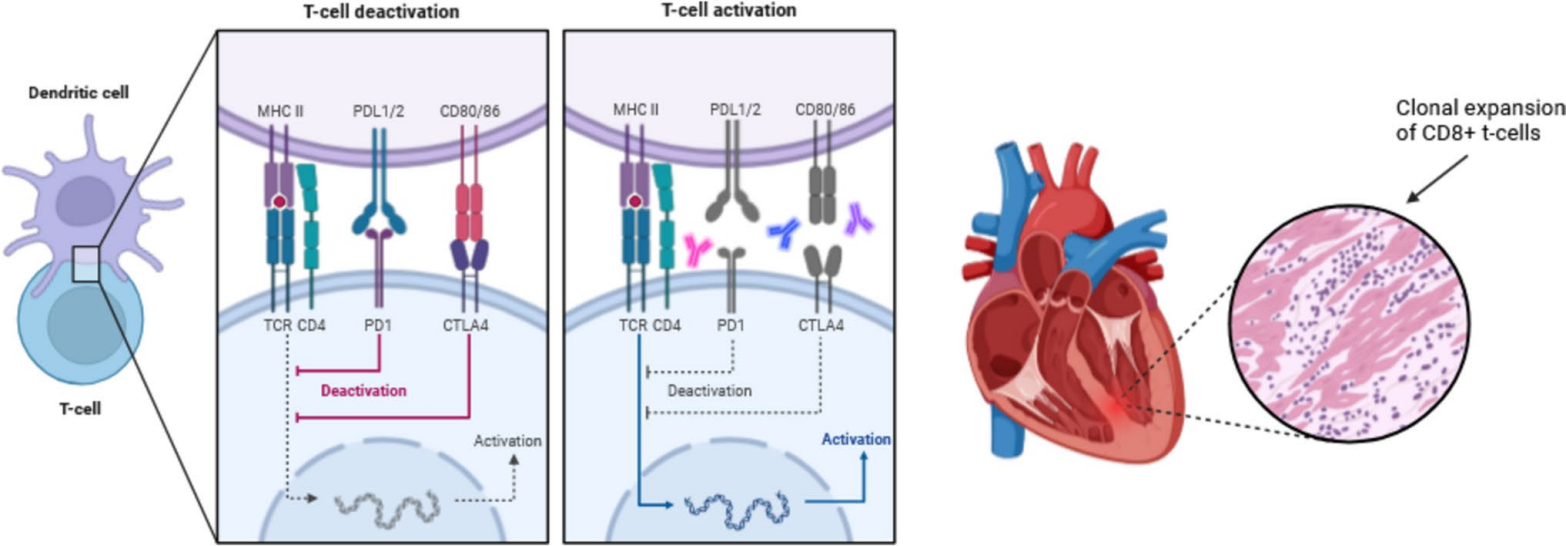
Schematic overview of immune checkpoint-inhibitor-related myocarditis. Modified from Seidel et al. [164]

Current preclinical models are primarily limited to global deletion of PD-1 in a gene-dosage dependent manner together with CTLA-4 [196]. However, development of spontaneous myocarditis is highly dependent on the background strain and the gender [22, 208]. In initial experiments, BLB/C mice have been shown to develop myocarditis when PD-1 was globally deleted, but the penetrance is highly variable in Bl6 mice [185]. ICI myocarditis is characterized by a clonal cytotoxic CD8 + cells expansion and infiltration in the myocardium [135, 210] (Fig. 2). Axelrod et al. identified a specific part of the alpha-myosin heavy chain (aa181–209 and aa406–425) as a candidate autoantigen which is recognized by specific T-cell clones that are enriched in the myocardium of patients having an ICI-myocarditis and yield new insights into ICI toxicity pathogenesis [14].

Despite the myocarditis, the use of ICIs can trigger other immune-related adverse events (irAEs), which may contribute to vascular inflammation and accelerate atherosclerotic plaque formation, thus increasing the risk of arteriosclerosis and cardiovascular complications [43].

Short-term ICI therapy in mice and also PD-1 deficiency induces T-cell-mediated plaque inflammation and drives plaque progression [30, 142]. Similar to the ICI-related myocarditis, CD8^+^ cytotoxic T cell numbers are an important key player in the immune response and accumulate in lymphoid organs and the arterial walls of the preclinical ICI model. Current evidence suggests that ICIs may accelerate plaque development and destabilization due to activation of pro-inflammatory cascades resulting in increased risk of cardiovascular events such as myocardial infarction and stroke in cancer patients. Atorvastatin is identified as a promising preventive treatment for ICI-related endothelial dysfunction and atherosclerosis in a mouse model. Nevertheless, further clinical studies are required for the establishment of the dual anti-PD-1 and atorvastatin therapy[45].

## Conclusion and outlook

Most current preclinical models investigating cardiotoxic drugs do not account for the co-existence of cancer. However, the toxicity of immune checkpoint inhibitors has shown that the presence of cancer is crucial for understanding cardiovascular pathophysiology. The selection of an appropriate model may also be influenced by the potential to combine it with genetic or pharmacological manipulation. Cardioprotective strategies must demonstrate that they do not interfere with the cytotoxic effects of cancer therapies which needs to be considered [189]. A comprehensive overview on the current therapeutic strategies and approaches is given by Moreno-Ariciniegas et al. [118].

In addition, we know from heart failure and post-myocardial infarction models, that there are sex-specific differences. While these differences are not yet well supported by data related to breast cancer, they are likely to exist. To identify and translate potential cardioprotective strategies, it will be essential to investigate cardiotoxic mechanisms in (1) suitable tumor models and (2) therapeutic strategies, complementary to those that are used in clinical studies in humans. In addition, this calls for a better preclinical characterization of existing tumor models and preclinical testing of novel cancer therapies. Our current understanding underestimates the accumulation of different events (pre-existing risk (genetic or epigenetic), combinational therapy, certain immunophenotypes and age [107]. Moreover, the strongest limitations come from the timepoint, which largely differs in many of the preclinical animal studies compared to the appearance in humans.
